# Five Year Analyses of Vegetation Response to Restoration using Rock Detention Structures in Southeastern Arizona, United States

**DOI:** 10.1007/s00267-022-01762-0

**Published:** 2022-12-19

**Authors:** Natalie R. Wilson, Laura M. Norman

**Affiliations:** grid.2865.9 0000000121546924 U.S. Geological Survey, Western Geographic Science Center, 520 N. Park Ave, Tucson, AZ 85719 USA

**Keywords:** Riparian restoration, Rock detention structures (RDS), Ecohydrology, Natural infrastructure in dryland streams (NIDS), Cienegas

## Abstract

Rock detention structures (RDS) are used in restoration of riparian areas around the world. The purpose of this study was to analyze the effect of RDS installation on vegetation in terms of species abundance and composition. We present the results from 5 years of annual vegetation sampling which focused on short term non-woody vegetation response within the riparian channel at 3 restoration sites across southeastern Arizona. We examined the potential ways that RDS can preserve native species, encourage wetland species, and/or introduce nonnative species using a Control-Impact-Paired-Series study design. Species composition and frequency were measured within quadrats and zones on an annual basis. Multivariate bootstrap analyses were performed, including Bray-Curtis dissimilarity index and non-metric multidimensional scaling ordination. We found that response to RDS was variable and could be related to the level of degradation or proximity to groundwater. The non-degraded site did not show a response to RDS and the severely degraded site showed a slight increase in vegetation frequency, but the moderately degraded site experienced a significant increase. At the moderately degraded site, located between two historic ciénegas (desert wetlands), species composition shifted and nonnative species invaded, dominating the vegetation increase at this location. At the severely degraded site, pre-existing wetland species frequency increased in response to the installation of RDS. These findings extend the understanding of RDS effects on vegetation, provide scenarios to help land and water resource managers understand potential outcomes, and can assist in optimizing success for restoration projects.

## Introduction

Riparian corridors, the area adjacent to a stream that contains specialized vegetation, are critical to the function of arid and semiarid landscapes. Ecologically, riparian areas increase overall biodiversity (Naiman et al. 1993; Sabo et al. 2005; Acuña et al. 2017), support habitat for rare species (Webb and Leake 2006; Caves et al. 2013; Minckley et al. 2013), and provide corridors for the movement of wildlife (Steward et al. 2012). In the western United States, riparian corridors are important for rangeland health (Whetstone 1994; Neary et al. 2004), serve as places of recreation, aesthetic enjoyment, and spiritual connection for local populations (Naiman et al. 1993; Patten 1998; Petrakis et al. 2020), and remain sacred sites to indigenous tribes (Whetstone 1994; Fox et al. 2017). Additionally, ciénegas, a specialized type of wetland, are associated with high quality riparian grasslands and intact ecohydrological processes (Hendrickson and Minckley 1984).

These valuable habitats are facing a variety of challenges. Historic wood-harvesting and overgrazing reduced vegetation, introduced non-native species, and compacted soils (Stromberg 1993; Richardson et al. 2007) while arroyo cutting severed the hydrological linkage between the channel and the floodplain, further decreasing riparian and ciénega habitats (Antevs 1952; Stromberg et al. 2007; Minckley et al. 2013). In recent decades, growing populations have increased water demand on both surface and groundwater supplies (Stromberg 2001; Webb and Leake 2006; Cornejo-Denman et al. 2020), and droughts are becoming more common and severe (Dominguez et al. 2010; Cayan et al. 2010; Ault et al. 2014). These issues combine to decrease water availability, which reduces vegetative cover, diversity, and primary productivity, and endangers the ecosystem functions and services provided by riparian corridors and associated ciénegas (Stromberg et al. 2007; Havstad et al. 2007; Norman et al., 2022b).

One of the semiarid regions of highest conservation concern in North America is the Madrean Archipelago or Sky Island region of the southwestern United States and northern Mexico. This region is globally recognized for its biodiversity and productivity (DeBano and Ffolliott 1994; López-Hoffman and Quijada-Mascareñas 2012; Devender et al. 2012). In 2013, concern for the unique biological, ecological, and cultural values of the Madrean Archipelago, including its riparian corridors and ciénegas, brought land managers, landowners, restoration practitioners, and scientists together to form the Sky Island Restoration Cooperative (SIRC, Norman et al. 2021, 2022a). This coalition seeks to “restore hydrologic and biologic processes throughout whole watersheds” (Buckley et al. 2014) and the members of the SIRC, with their combined experience and expertise, began developing restoration projects to protect this landscape.

The restoration of watershed hydrology is a pre-requisite for meaningful restoration in riparian systems (Palmer et al. 2010). In arid and semiarid regions, where vegetation dynamics are primarily water-driven (Horton et al. 2001; Nemani et al. 2003; Stromberg et al. 2007), changes in hydrological dynamics should affect vegetation dynamics and the restoration of hydrologic processes should support the natural regeneration of riparian vegetation composition and structure (Stromberg et al. 2007; White and Stromberg 2011). In the Madrean Archipelago, a review of research conducted on RDS in the 1960s found that they were often breached, and those that remained varied in their effect on vegetation cover (Baker et al. 1994). Later research in the Madrean Archipelago focused on water supply and erosion, with little analysis of effects of altered hydrology on vegetation (DeBano and Ffolliott 1994).

Since 2013, U.S. Geological Survey (USGS) scientists have been studying aridland water harvesting approaches to restoring productive landscapes and increasing climate resilience by quantifying the effects of RDS on hydrologic, geomorphic, and biologic processes in the Madrean Archipelago as part of the Aridlands Water Harvesting Study (http://usgs.gov/WGSC/Aridlands/, Norman 2020). Research in collaboration with the SIRC has demonstrated positive effects on hydrological parameters across multiple temporal scales, from the first year after the installation of RDS to decades later. These changes include reduced peak flows, increased infiltration, increased sediment deposition and reduced transport, and an elongated season of flow (Norman et al. 2015; 2017; 2019; Norman and Niraula 2016). To examine the effect of these hydrologic changes on riparian vegetation, remote sensing techniques were applied to analyze mature restoration sites, where RDS were installed more than 30 years ago, resulting in the determination that RDS increased vegetation greenness and health at RDS locations, in times of drought, and extended as far as 5 km downstream of RDS (Norman et al. 2014; Wilson and Norman 2018). This finding supports the link between restoration of hydrologic processes and riparian vegetation health but only examined general vegetation condition response and trends on a decadal scale, with limited investigation of plant response at a species level.

Based on anecdotal evidence at mature RDS restoration sites, combined with ecological concepts such as succession theory and documented hydrological response, we hypothesize that due to increased water availability, vegetation response at RDS will include increases in abundance of vegetation and in species richness. Through involvement in the SIRC, our connections with restoration practitioners, landowners, and land managers, allowed us the opportunity to study watershed restoration projects as they were being implemented. In this paper we present the quantitative results from the short-term vegetation response research which consisted of annual sampling for 4–5 years at 3 different sites using several types of RDS in in the Madrean Archipelago.

## Study Area and Project Sites

The Madrean Archipelago has a distinct and diverse assemblage of species due to the overlap of multiple intersections of biogeographical characteristics (Felger and Wilson 1994; McLaughlin 1994; Coblentz and Riitters 2004). Also known as the Sky Island region, it has a complex topography due to being part of the Basin and Range Province where tall mountain chains are separated by low arid valleys. It lies between two biotic provinces, the temperate Rocky Mountains to the north and the neotropical Sierra Madre Occidental to the south. Finally, this region inhabits the transition zone along the continental divide between the Sonoran Desert to the west and the Chihuahuan Desert to the east. This juxtaposition creates a unique assemblage of vegetation communities organized by a combination of climatic, geographical, and topographical factors (Baker et al. 1994; Felger and Wilson 1994). Mixed coniferous forests inhabit the highest, wettest mountain peaks while the lowest, driest valley floors are home to either Chihuahuan or Sonoran Desert scrub, dependent on the east-west variations in soil type and precipitation patterns. Just higher than the lowest desert valleys are semidesert grassland. These grasslands extend up in elevation, transitioning through savanna and into mixed oak-conifer woodlands. Precipitation in the region historically follows a bimodal pattern; summer storms during the monsoon season are sudden, short, fierce, and provide most of the annual rainfall while winter storms are generally gentle, long and provide snowfall in the higher elevations (Phillips and Comus 2000).

**Fig. 1:**
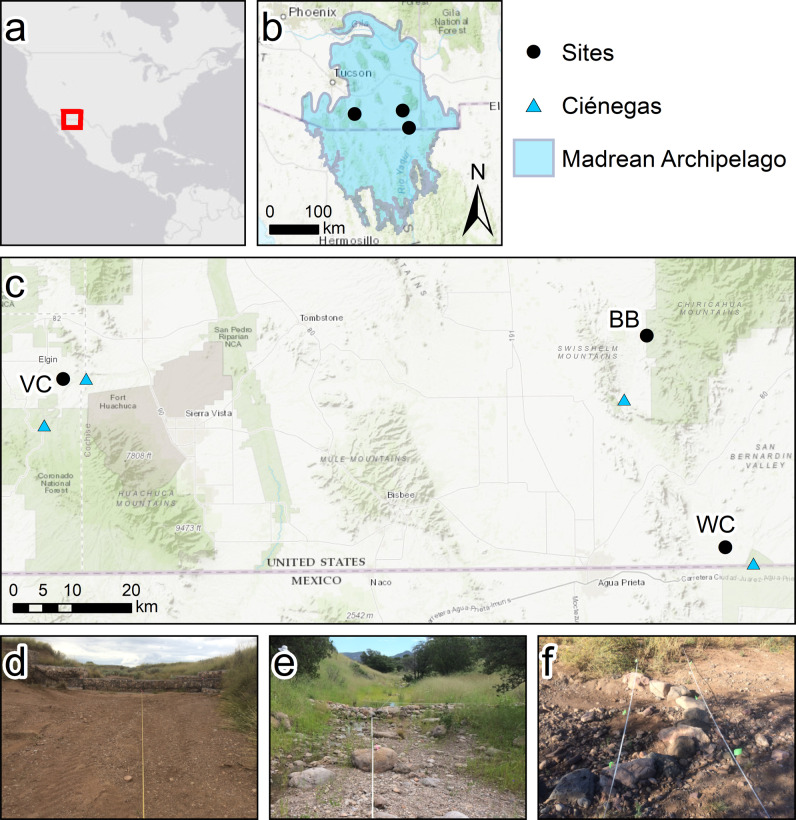
Location of study sites and examples of rock detention structures (RDS). **a** Inset map showing location of the Madrean Archipelago within North America. **b** Study sites within the Madrean Archipelago in the United States-Mexico region. **c** Study sites and noted ciénegas within southeastern Arizona. VC Vaughn Canyon, BB Barboot, WC Wildcat Canyon. **d** Gabion at Vaughn Canyon, looking upstream at the RDS. **e** Check dam at Barboot looking upstream. **f** Rosgen cross vane at Wildcat Canyon, the right side is upstream with flows going to the left. USGS photos by N. R. Wilson. Base map by ESRI et al. (ESRI et al. 2021a, b)

To evaluate the effects of RDS on vegetation, 3 project sites were established in 2015 shortly after RDS were installed. Our study sites extended across southeastern Arizona (Fig. 1) and included a variety of vegetation communities, hydrological characteristics, and other ecologically pertinent characteristics (Table 1). Land ownership also varies between sites, leading to the involvement of different stakeholders at each study site, including different restoration practitioners, and practices, for each site. Restoration practitioners involved at each project site performed site evaluations and applied site-specific nature-based solutions to address goals identified by project managers, with consideration to the physical characteristics of a site (topography, hydrology, vegetation, and degradation) and the resources available (labor costs, and material cost/availability). The approaches ranged from small, hand installed rock structures, to boulder structures installed as part of a large land sculpting effort, but all were undertaken as actions “… designed to assist natural processes of recovery that ultimately are carried out by the effects of time on physical processes and the responses and interactions of the biota throughout their life cycles.” (Gann et al. 2019).

**Table 1:** Site characteristics. Temperature and precipitation are averaged over the study period (2015 – 2019)

Site	Barboot	Vaughn Canyon	Wildcat Canyon
Elevation	1570 m	1420 m	1185 m
Jan Temp Range	0.3–14.5 °C	−1.9–16.5 °C	2.4–17.5 °C
Jun Temp Range	15–32.1 °C	14.4–34.4 °C	26.5–36.6 °C
Annual Precipitation	44.3 cm	43.3 cm	37.8 cm
Monsoon Precipitation (July – Sept)	23.5 cm	26.1 cm	21.7 cm
Soils	Lemitar-Lampshire-Chiricahua association, well-draining	Grabe soils, well-draining	Guest-Riverroad association, well-draining
Depth to Water Table	--^a^	> 2 m	> 2 m

For all sites, temperatures were consistent with previous 20 years and both annual and monsoon precipitation, excluding 2017, was slightly higher than previous 15 years. 2017 annual precipitation was lower, similar to the previous years, but the monsoon was comparable to other years of the study period. (Farr et al. 2007; Lawrimore et al. 2016; Soil Survey Staff, Natural Resources Conservation Service, United States Department of Agriculture 2021; PRISM Climate Group 2022)

^a^No information was available for depth to water table at Barboot

### Barboot

The Barboot grazing allotment (Barboot) lies in the southern Chiricahua Mountains in the Coronado National Forest (BB, Fig. 1). Naturally occurring vegetation includes a mixed-oak, juniper tree savanna/woodland, with canopy cover increasing along drainages and on wetter north and east facing slopes. Barboot was the least-degraded site with little evidence of grazing despite the occasional presence of cattle. Barboot drains a section of the western slopes of the Chiricahua Mountains. Channel morphology varied throughout the study area with channel widths ranging from 3 m in upstream plots to 10 m further downstream. Banks varied based on surrounding topography, with some low banks leading to broad floodplains and others being steep bedrock hillslopes. Below the National Forest are private ranches that extend down the lower slopes of the Chiricahua Mountains and flank the drainage as it turns south between the Chiricahua and the Swisshelm Mountains. Here, 11 km downstream from Barboot, is the Leslie Canyon Ciénega, just before the drainage passes through the Leslie Canyon National Wildlife Refuge (Hendrickson et al. 2021). While it was the least-degraded site in terms of habitat and vegetation community, the whole Madrean Archipelago has been affected by climate change and forest management histories resulting in increased risks of catastrophic wildfire (Villarreal and Yool 2008; Coe et al. 2012). One such fire, the Horseshoe 2 Fire, burned over 70% of the Chiricahua Mountains in 2011 (Youberg et al. 2012); as one of the remaining unburned areas, the purpose of the project at Barboot was to increase resilience to future wildfire (Campbell and Misztal 2015). Restoration consisted of over 100 RDS placed along the main reach and four additional tributaries. RDS installed were predominately small check dams, loose rock structures placed perpendicular to the direction of flow. RDS were installed in the summer of 2015, with some repairs and maintenance performed in 2016. Plots were located along a 1.4 km section of the main drainage as well as three different tributaries. In 2019, additional RDS from a prior, undocumented restoration project were found upstream of the 2015 project area.

### Vaughn Canyon

Vaughn Canyon contributes to the upper Babocomari River near Elgin, Arizona (VC, Fig. 1). Vegetation is sacaton grassland with remnants of a previously extensive cottonwood – willow forest persisting in small, scattered groups of trees along the channel. This site often had cattle present and evidence of recent grazing, but the main indication of degradation was the wide arroyo, an incised dryland stream, that separated the channel and the current floodplain from the higher historic floodplain. Vaughn Canyon drains into the Babocomari Ciénega 3.8 km downstream of the restoration, where the canyon joins the Babocomari River; 8 km to the south, in neighboring O’Donnell Canyon is the Canelo Hills Ciénega (Hendrickson et al. 2021). The Babocomari River drains to the east, and is a tributary of the San Pedro River, a critical source of water and habitat in the region (Yuncevich 1993). The purpose of restoration at Vaughn Canyon was to restore hydrological function by increasing infiltration and aquifer recharge (Norman et al. 2019). Restoration consisted of 5 large gabions, installed with heavy machinery, within the large arroyo. The sides of the arroyo are 2 to 3 m tall and the bottom ranges from 8 to 13 m wide. It is through this broad bottom that a secondary channel meanders, with banks generally <0.5 m tall and widths of 8 to 14 m. Upstream, a variety of techniques were used to address the head-cutting and other erosional concerns. The gabions and some upstream RDS were initially installed in 2015. Construction of upstream RDS continued to 2017. Due to heavy rains in 2015, two of the gabions required repair in 2016; all gabions held into the 2017 season.

### Wildcat Canyon

Wildcat Canyon flows southeast and drains into Silver Creek approximately 25 km east of Douglas, Arizona and 3 km north of the US-Mexico border (WC, Fig. 1). Vegetation is remnant sacaton riparian grassland with a xeric mesquite burrobrush channel. It was, qualitatively, the most degraded site with obvious gullying, low grass cover on the historic sacaton floodplain, and heavy shrub encroachment around the active channel and on the floodplain. After Wildcat Canyon joins Silver Creek, Silver Creek continues south across the border, trending southeast to join the Rio San Bernardino 2 km south of the international border and the southern edge of the San Bernardino National Wildlife Refuge. From 1984–2012, the U.S. Fish and Wildlife Service did extensive restoration on other tributaries of the Rio San Bernardino north of the border, ~5 km from Wildcat Canyon, to revitalize the Ciénega San Bernardino and associated fish habitats in the riparian corridor. South of the border, 3 km downstream of Wildcat Canyon, Silver Creek enters Rancho San Bernardino, owned and operated by Cuenca los Ojos which has also done extensive restoration on the San Bernardino ciénega, installing hundreds of RDS since 2001 (Norman et al. 2014; Wilson and Norman 2018). The goal of restoration at Wildcat Canyon was to reduce the intensity of flows and improve the condition of the riparian grassland (Jeff Conn, Bureau of Land Management, oral communication, 11/20/2015). Restoration was primarily a “Plug ‘N Pond” style of restoration where the old channel was “plugged” with earthen dams to create “ponds”, and the flow redirected into new meander channels along the flood plain (Hammersmark et al. 2010; Zeedyk and Clothier 2014). Four plugs were built and ~300 m of new channel was created on the floodplain separating Wildcat Canyon and Silver Creek. The original width of the channel ranged from 2 to 4.5 m while the new channel is 3 to 4 m wide. Rosgen cross vanes are a type of one-rock dam created by installing one layer of rocks in a U-shape (Fig. 1, Rosgen 2011); eight of these Rosgen cross vanes were built in the new channel and four additional ones were built in the original channel below the Zuni bowl. Small plugs of a native bunchgrass, *Sporobolus wrightii* (giant sacaton), were planted. Restoration was completed in March 2015. Due to heavy precipitation in July 2015, totaling 10.9 cm for the month, 5 cm of which fell during a 24-hr period on 7/23/15 (Station ID: WBAN:93026, Lawrimore et al. 2016), heavy flows damaged the RDS, one was completely destroyed, and most of the vegetation plugs were washed away. January of 2016, repairs were made to the RDS and channel, and the restoration practitioner transplanted large sections of sacaton (estimated 600 plants) from the floodplain into the new channel to slow flows. Plots were placed along a ~650 m section of the drainage between the confluence with Silver Creek downstream and a change in landownership upstream. This section of Wildcat Canyon consists of a channel with a broad floodplain to the east and a steep bank to the uplands to the west. The channel is cut ~1.5 to 3 m down from the flood plain on river left and higher on river right.

## Methods

To guide restoration project assessment, we used the Society of Ecological Restoration method (Society for Ecological Restoration International Science & Policy Working Group 2004; Ruiz-Jaen and Mitchell Aide 2005), which identifies 3 major ecosystem attribute categories of importance: vegetation structure, diversity, and ecological processes. To allow for annual revisits for several years, we focused on measuring only vegetation structure and diversity. However, by collecting data over several years and directly assessing community dynamics of colonization and succession, we are indirectly assessing the ecohydrological processes associated with water availability.

### Sampling Design

We use the term “site” to refer to the different restoration project locations; “plot” refers to either a control or treatment location where we collected frequency and composition data in quadrats and zones. Our experimental design was based on a Before-After-Control-Impact-Paired-Series (BACIPS) design which requires impact and control sites to be sampled multiple times before and after intervention, the installation of RDS in our case. However, we were not able to collect data prior to RDS installation at any site, necessitating a modified “Control-Impact-Paired-Series” design (Underwood 1994; Thiault et al. 2017). We located treatment plots at RDS and identified matched control plots in the same drainages, up and downstream from RDS. When identifying control plots, the scale of the possible effect of an RDS was a concern (Underwood 1994; Norman et al. 2014; Wilson and Norman 2018). To reduce the possibility of RDS effects on our control sites, we excluded areas around a structure. The size of the area excluded was based on the size of the RDS and inferences from other restoration projects. This area was 50 m at Barboot and 100 m at Vaughn Canyon. At Wildcat Canyon the control sites were located at least 30 m upstream or downstream of any RDS or other restoration activity. The number of treatment and control plots installed at a site was based on the general size of the restoration project, overall number of RDS, and the amount of time available to collect data. The presence of tall woody plants or a narrow channel would preclude plot installation, in which case, that plot location was rejected and the next random plot location used. For ease of location during revisits, all plot locations were recorded with a Garmin GPMAP 62sc GPS unit at the center of the plot; additionally control plots were marked with rebar.

### Sampling Methods

**Fig. 2:**
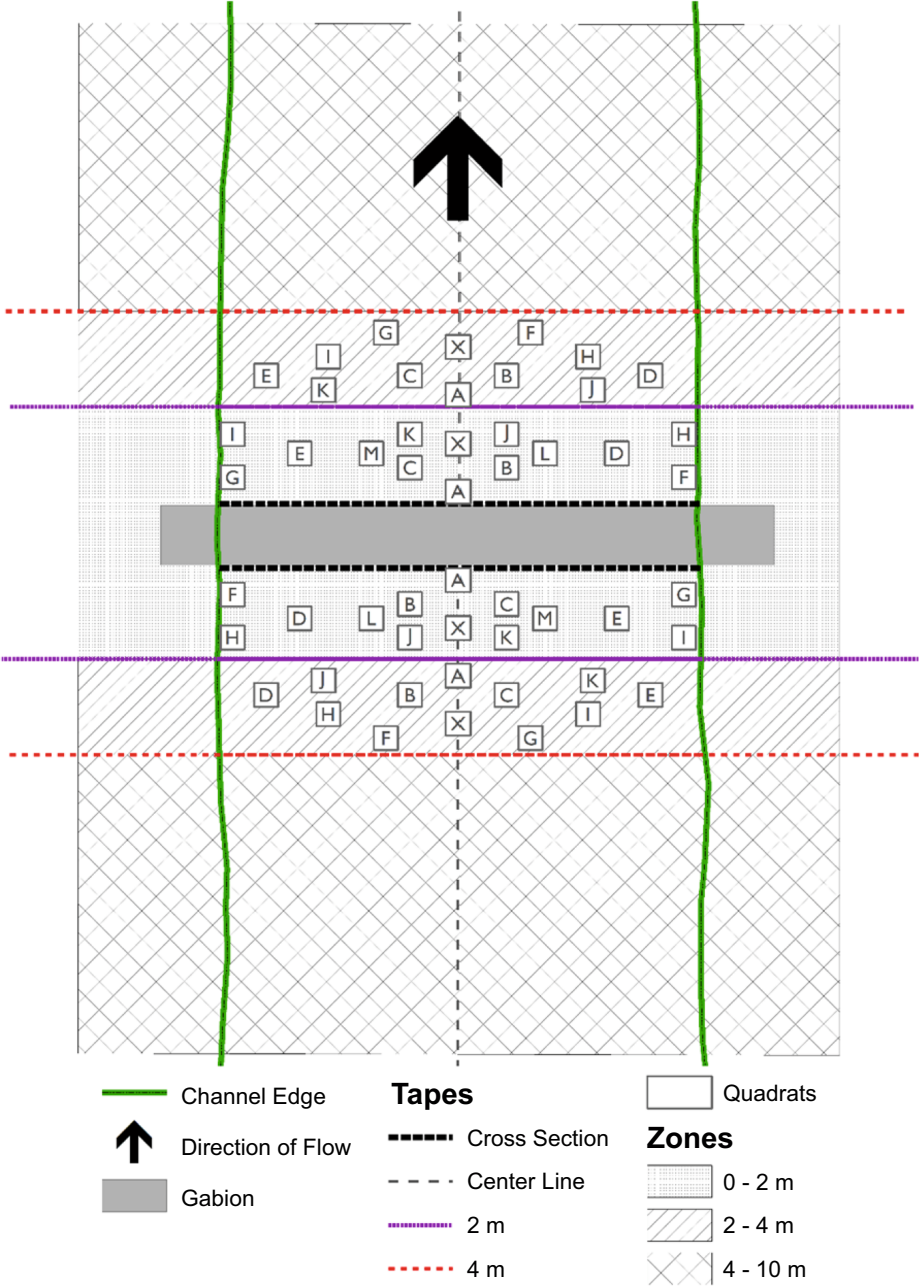
Generalized diagram of the study plot design at a RDS, gabion type, in a 10 m wide channel. For a control plot, the RDS would be represented by a single cross section tape, effectively an RDS with 0 depth along the channel. Tapes were used to delineate zones: upstream 0–2 m, upstream 2–4 m, upstream 4–10 m, and similar for downstream. Zones extended onto the bank ~3 m. The location of quadrat A within the 0–2 m zone was based on the intersection of the center tape and the cross section, within the 2–4 m zone the location was based on the intersection of the center line and the 2 m tape. Other quadrat locations were systematically randomized based on orthogonal distances from the location of quadrat A. Quadrats were sampled in alphabetical order. If the channel was <10 m wide, then locations that fell outside the channel were not sampled. Species presence/absence as well as basal and foliar cover was recorded within the quadrat (Elzinga et al. 1998)

For our measurement of vegetation structure, we measured frequency of species using quadrats combined with visual estimates of foliar and basal cover using a modification of the Domin-Krajina scale (Krajina 1933). At treatment plots, RDS were generalized to a quadrilateral shape using transect tapes. The plots were stratified by their position instream (i.e., upstream or downstream of the RDS) and by proximity to the RDS (0–2 m, 2–4 m) creating six zones per plot (Fig. 2). For control plots, a cross-tape was laid perpendicular to the channel to represent a RDS with a depth of 0 m and then the plot was divided in the same manner as treatment plots. The center of the RDS or channel was determined and a transect tape was laid from this point along the middle of the channel for at least 10 m along the length of the channel, parallel to direction of flow. This created a pseudo-Cartesian grid on the location, though at some plot locations the grid was skewed due to the meander of the channel. This grid formed the origin point for the quadrat locations. Possible quadrat locations were systematically randomized within each zone based on a 10 m wide channel and quadrat locations were attempted in alphabetical order (Fig. 2). Any quadrat location which would put 25% of the quadrat outside of the channel edge was excluded and the next location attempted. A minimum of two quadrats per zone were required to sample a site; a maximum of seven quadrats per zone were sampled. Quadrats were 0.5 m × 0.5 m, constructed of PVC with string to divide the full quadrat into smaller quadrats nested within the larger one; smaller nested quadrat measurements were 0.5 m × 0.25 m, 0.25 m × 0.25 m, 0.25 m × 0.125 m, and 0.125 m × 0.125 m. Species were recorded in the smallest nested quadrat they occupied. All quadrats were photographed each year and photos of the entire plot were taken, one from upstream of the RDS/control location and one from downstream (Wilson et al. 2021). Species data were collected for the zones defined by the tapes within the channel as well as on the bank up to 3 m outside of the channel edge to provide qualitative data for each site (Fig. 2).

This methodology was developed to capture the first vegetation response we expected to observe, herbaceous vegetation within the channel. However, in semiarid systems annual species are highly sensitive to both inter- and intra-annual variations in precipitation while perennial species can withstand these temporal variations (Chesson et al. 2004; Reynolds et al. 2004; Sher et al. 2004). Consequently, we only identified perennial plants to species, and most annual species were identified to life-form (i.e., forb, grass, sedge), emphasizing their function in the ecosystem. Suspected non-native plants were identified to species independent of duration, and data were collected by species. Plants that could not be identified but were suspected to be perennial, non-native, or wetland species were collected and identified later. We chose to sample during the middle of the summer monsoon season (August and September) because it is the strongest of the two growing seasons in the Madrean Archipelago (Dimmitt 2000; Sheppard et al. 2002; Wilson et al. 2016). Sampling occurred from 2015 to 2019.

### Plant List

A master plant list was developed by integrating data from several national resources with resources developed by experts in the vegetation of southeastern Arizona (U.S. Army Corps of Engineers 2012; Buckley 2015a, b; SEINet 2015; NRCS USDA 2015). This list documents taxonomic data as well as data on life form, duration, non-native status, wetland status, and conservation concern. The latter three variables were restricted to binary data for our analysis. To be considered a wetland species for our analysis, the species had to be considered either wetland obligate (almost always occurs in wetlands) or a wetland facultative (usually occurs in wetlands) in the National Wetland Plant List (NWPL; U.S. Army Corps of Engineers 2012). Other species, primarily facultative species according to the NWPL, species that occur equally in wetland and upland areas, were included as wetland species based on local ecological knowledge (Buckley 2015a, b). This plant list, and more detailed information on its development, can be found in the USGS data release corresponding with this paper, (Wilson et al. 2021).

### Analysis

Dissimilarity indices are a way to quantify the difference between vegetation communities in plots. The Bray-Curtis index is a widely used quantitative dissimilarity coefficient that provides ecologically relevant information and is suited to situations where environmental heterogeneity is low and the difference between samples is due to differences in treatments or short-term temporal changes (Bray and Curtis 1957; Tamás et al. 2001; Ricotta and Podani 2017). Nonmetric multidimension scaling (NMDS) is a commonly used ordination method in vegetation analysis based on the matrix output of a dissimilarity coefficient analysis such as the Bray-Curtis index (Legendre and Legendre 2012). Both are widely used to evaluate the effectiveness of restoration (Wilkins et al. 2003; Matthews and Spyreas 2010; Merdas et al. 2021). Both the Bray-Curtis index and NMDS were applied to perennial vegetation at plots by site and across all sites. Bray-Curtis dissimilarity was used to compare intragroup variation within treatment/control plots as well as the variation between treatment and control plots. NMDS was calculated for plots between the first and last year of sampling in the same groupings. Finally, the frequency of total perennial, annual, non-native, and wetland vegetation was calculated for each plot and visualized by site through the years (Elzinga et al. 1998).

Community analysis generally assumes equal area (Bonham 1989), but our sampling protocol was developed to accommodate the variability of hydrological systems, allowing for flexibility when applying the protocol to channels of different widths as well as change in channel dimensions over time. This results in different numbers of quadrats at each plot and different numbers of quadrats at the same plot in different years, creating variability in sampling area per plot. To control for this, the minimum number of quadrats sampled at each plot for the full sampling period was determined for each site (Table 2). This minimum number determined the number of quadrats subset from each plot to control for sampling area in analysis. However, if a plot had more than the minimum number of quadrats, a single subset of quadrats at that plot would not capture the full variability of that plot. To capture this variability, bootstrapping was applied with 500 iterations for each analysis. Bootstrapping is a statistical method that subsets the data in a randomized and iterative manner to allow for the application of statistical measures of accuracy that cannot be calculated on a single sample (Efron 1979). For dissimilarity analysis and frequency analysis, these iterations were combined for the analyses which allowed us to calculate confidence intervals. For the NMDS analysis, an ordination was computed and graphed for each iteration and general patterns analyzed (Legendre and Legendre 2012).

**Table 2:** Sampling effort by site

Site	Sampling Years	Control Plots	Treatment Plots	Minimum Number of Quadrats per Plot	Maximum Number of Quadrats per Plot
Barboot^a^	2015–2018	6	5	9	20
Vaugh Canyon	2015–2019	2	2	14	28
Wildcat Canyon^b^	2015–2019	4	4	12	22

^a^At Barboot, 13 plots were installed in 2015 but 2 plots were abandoned in 2016 as their RDS had been destroyed by damaging flows; the data from those plots were excluded from analysis

^b^Wildcat Canyon had 6 plots installed in 2015. In 2016, 2 more control plots were added. In the same year, one treatment plot could not be relocated due to flows damaging the RDS. A nearby RDS was mistaken for the original plot and sampled. Data from the original and the “imposter” plot were comparable and combined for this analysis

### Data Workflow

Data were entered on paper in the field then transcribed to a Microsoft 365 Excel spreadsheet every year. The yearly data was appended to a Microsoft 365 Access relational database which contained the data for all years of the study (Wilson et al. 2021). Data integrity checks were completed in Access. For analysis, data was exported to CSV and analyzed in R v4.1.2 in RStudio v2021.09.2 (R Core Team 2021; RStudio Team 2022). Additional data quality checks were completed in R before analysis. R packages used include data.table, ggplot2, reshape2, and vegan (Wickham 2020; Oksanen et al. 2020; Wickham et al. 2021; Dowle and Srinivasan 2021). Spatial point data was collected at each plot with a hand-held GPS and imported into an ESRI ArcGIS geodatabase using dnrgps v6.1.0.6 (Minnesota Department of Natural Resources 2014; Wilson et al. 2021).

## Results

Total number of plots installed, the number of treatment and control plots, the maximum and minimum number of quadrats per plot, and sampling years for each site can be found in Table 2. No plot locations were excluded based on the disqualifying conditions of pre-existing tall woody vegetation or narrow channel width. When considering the nested quadrat data, we determined the largest quadrat size (0.5 m × 0.5 m) was the most sensitive for the frequencies found at our study sites (Smith et al. 1987; Elzinga et al. 1998) and it is the only size used in the following analysis. Within these quadrats, foliar cover correlated with frequency unless plots had been grazed. When grazed, frequency provided a more stable measure. This agrees with the general consensus on the strengths and weaknesses of these two metrics (Elzinga et al. 1998; Carlsson et al. 2005). As such, our statistical analysis focused on frequency as the metric of vegetation abundance. Some species were difficult to identify in the field at the time of observation; instead of collecting a sample from each individual, these species were combined into species complexes. Complexes include the *Schizachyrium cirratum/sanguineum* complex, *Cyperus pallidicolor/hermaphroditus* complex, and the *Setaria leucopila/macrostachys* complex. These were counted as single species in analysis. All results can be found in the USGS data release corresponding with this paper (Wilson et al. 2021).

### Species Composition

Fifty-four species were observed in quadrats at all sites. At all sites, Poaceae was the most dominant family, though dominant genera varied by site. At Barboot, *Bouteloua* and *Muhlenbergia* were the most common genera while at Vaughn Canyon *Sporobolus* was the most common. Wildcat Canyon had no dominant Poaceae genera. Asteraceae was well represented at all sites but at Barboot Fabaceae and Cyperaceae were also common. Overall, Barboot was the most diverse site with 32 perennial species observed in the quadrats; 14 species were observed only at Treatment plots and 4 species were observed only at Control plots. At Vaughn Canyon, 20 perennial species were observed in the quadrats; 14 species were observed only at Treatment plots and no species were observed only at Control plots. At Wildcat, 15 perennial species were observed in the quadrats 9 species were observed only at Treatment plots and 2 species were observed only at Control plots. Of the 2475 observations in quadrats, only 12 remained unidentifiable. Perennial species observed in quadrats at each site can be found in supplementary materials, Table S1.

Additional species were noted in the zones and are presented to provide a qualitative account of each site, see supplementary materials, Table S2. One hundred and ninety-seven species were observed at all sites. The dominance of families was consistent with quadrat results. Barboot remained the most diverse site with 129 perennial species observed in the zones; 18 species were observed only at Treatment plots and 37 species were observed only at Control plots. At Vaughn Canyon, 73 perennial species were observed in the zones; 18 species were observed only at Treatment plots and 17 species were observed only at Control plots. At Wildcat, 58 perennial species were observed in the zones; 18 species were observed only at Treatment plots and 16 species were observed only at Control plots. Additionally, there were six wetland annual species, six nonnative annual species, and one nonnative wetland annual species observed at the project sites. No threatened or endangered species were observed but seven species that are salvage or harvest restricted in the state of Arizona were present. Of the 8124 observations in the zones, only 76 remained unidentifiable.

### Frequency Analysis

**Fig. 3:**
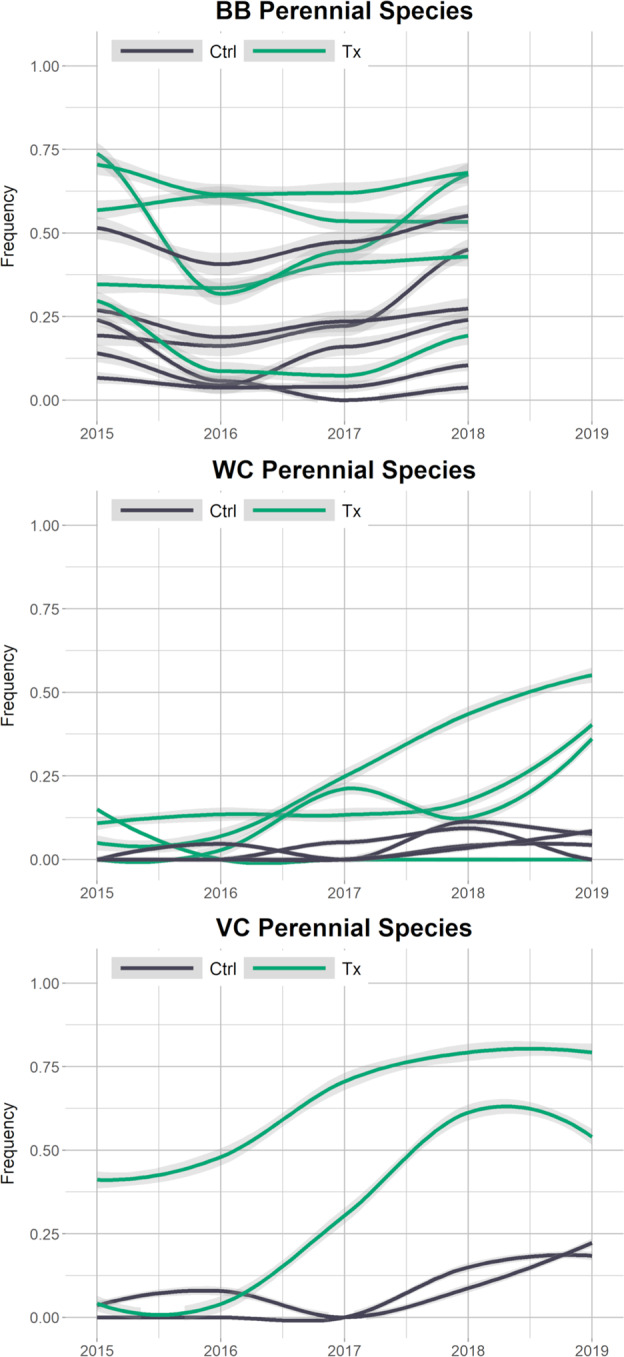
Frequency of perennial vegetation by plot over time with bootstrap iterations. Loess smoothing, 95% confidence interval as shaded gray. Top: Barboot (BB); Middle: Wildcat Canyon (WC); Bottom: Vaughn Canyon (VC). Ctrl control, Tx treatment

The change in the frequency of perennial vegetation over time differed between study sites (Fig. 3). Vaughn Canyon showed the clearest increase in perennial vegetation at the treatment plots compared to control plots. One treatment plot and both control plots had low frequencies of vegetation during the first year of observation (2015), the other treatment plot had a greater abundance of perennial vegetation. At Wildcat Canyon all plots had similarly low frequencies of perennial vegetation the first year after restoration. Over the study period, 3 of 4 of the treatment plots had a moderate increase in perennial vegetation while one treatment plot and all control plots continued to have low abundances of perennial vegetation. At Barboot, the frequency of vegetation at both treatment and control plots was highly variable in the first year of the study and this variability continued through the study period with little difference discernable between treatment and control plots over time.

**Fig. 4:**
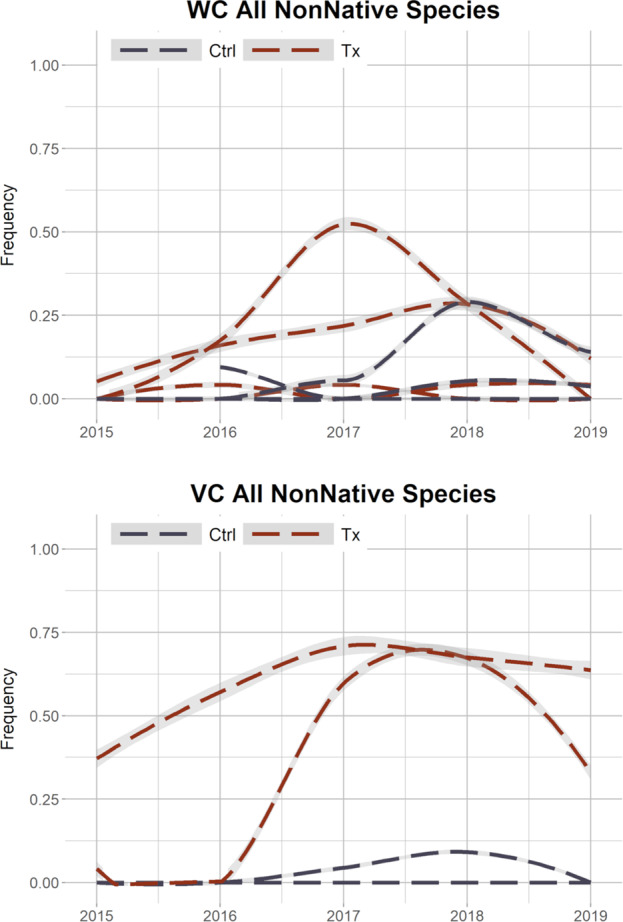
Frequency of nonnative vegetation, perennial and annual, by plot over time with bootstrap iterations. Loess smoothing, 95% confidence interval as shaded gray. Top: Wildcat Canyon (WC); Bottom: Vaughn Canyon (VC). Ctrl control, Tx treatment

The most common wetland species at Wildcat Canyon and Vaughn Canyon were *Cyperus esculentus* (yellow nutsedge) and *Sporobolus wrightii* (giant sacaton) and both were major contributors to the increase in perennial vegetation frequency at treatment plots over time at those sites (Figure S1). At Barboot, *Echinochloa crus-galli* (large barnyard grass), an annual, and *C. esculentus* were the most common wetland species but no clear trends were evident due to the variability over the project site during the study period (Figure S1). Nonnative species were similarly variable in plots over time at Barboot. However, both Wildcat Canyon and Vaughn Canyon had notable trends in nonnative vegetation over time (Fig. 4). At Wildcat Canyon, at 2 treatment plots, nonnative vegetation increased over the first 3–4 years then decreased the 5th year. At Vaughn Canyon, two perennial nonnative grasses, *Cynodon dactylon* (Bermuda grass) and *Sorghum halepense* (Johnson grass), contributed to the increase in perennial vegetation at treatment plots over time.

### Community Analysis

**Fig. 5:**
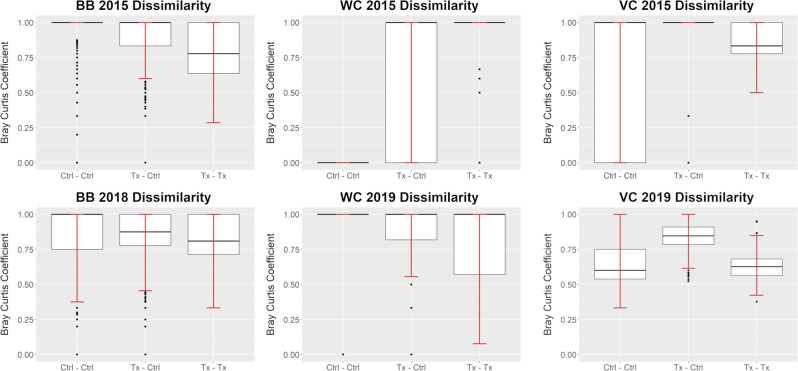
Bootstrapped (500 interactions) Bray-Curtis dissimilarity analysis applied intragroup for control plots (Ctrl-Ctrl) and treatment plots (Tx-Tx), and intergroup (Tx-Ctrl) for the first and last year of sampling at all sites. A value of 1 is perfectly dissimilar and a value of 0 is perfectly similar. Left: Barboot (BB); Middle: Wildcat Canyon (WC); Right: Vaughn Canyon (VC). Top: First year of sampling; Bottom: Last year of sampling. Barboot was not sampled in 2019

Typically, in dissimilarity analysis, empty plots are excluded since the absence of species doesn’t indicate any meaningful ecological similarity for those plots (Legendre and Legendre 2012). However, in analyzing drainage channels, empty plots are common. These “double zeros” were retained to indicate an absence of perennial vegetation. This resulted in many extreme dissimilarity values, including both perfectly similar, a value of 0, and perfectly dissimilar, a value of 1, results (Fig. 5).

At Barboot, in 2015, the median intergroup, treatment vs control plots, dissimilarity was 1 and the interquartile range (IQR) was 0.17. Median intragroup dissimilarity for control plots was 1 with an IQR of 0. Median intragroup dissimilarity for treatment plots was 0.78 with an IQR of 0.36. In 2018, control and treatment plots had become more similar with a median intergroup dissimilarity of 0.88 and an IQR of 0.22. Median intragroup dissimilarity for control plots was 1 with an IQR of 0.25 while intragroup dissimilarity for treatment plots remained close to 2015 values with a median of 0.81 and IQR of 0.29.

At Wildcat Canyon, in 2015, the median intergroup, treatment vs control plots, dissimilarity was 1 and the IQR was also 1; all plot combinations returned extreme values of 1 or 0. Median intragroup dissimilarity for control plots was 0, all plots were perfectly similar due to a total lack of perennial vegetation. Median intragroup dissimilarity for treatment plots was 1 with an IQR of 0, though some non-extreme values did occur. In 2019, control and treatment plots had become less similar with a median intergroup dissimilarity of 1 and IQR of 0.18. Median intragroup dissimilarity for control plots was 1 with an IQR of 0, all results were extreme values of 0 or 1. Treatment plots became more similar in 2019 with a median intragroup dissimilarity of 1 and a IQR of 0.43.

At Vaughn Canyon, in 2015, median intergroup, treatment vs control plots, dissimilarity was 1 with a IQR of 0. Median intragroup dissimilarity for control plots was 1 with an IQR of 1, all results were extreme values. Median intragroup dissimilarity for treatment plots was 0.83 with an IQR of 0.22. In 2019, median intergroup dissimilarity was 0.85 with an IQR of 0.12. Both treatment and control plots saw lower levels of intragroup dissimilarity. Median intragroup dissimilarity for control plots was 0.60 with an IQR of 0.21 and for treatment plots it was 0.63 with a IQR of 0.12.

**Fig. 6:**
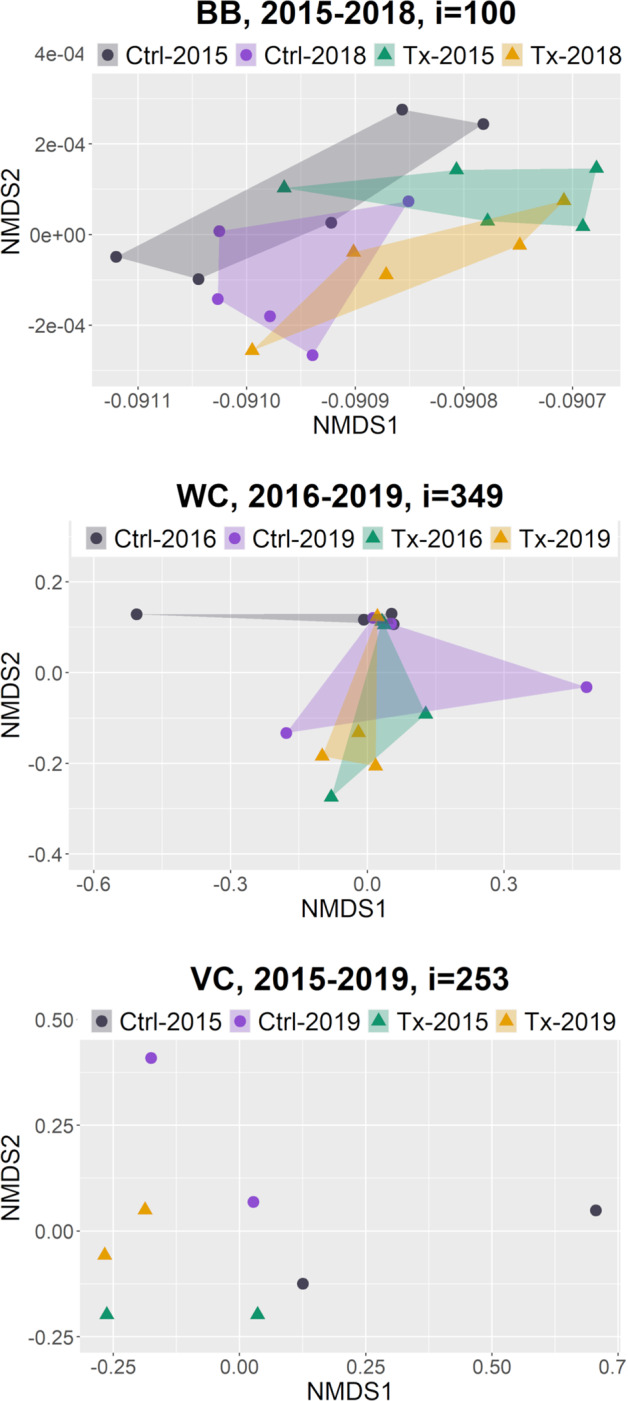
Representative nonmetric multidimension scaling (NMDS) ordinations comparing treatment and control plots for different years. Top: Barboot (BB), first vs last year with 2 outliers removed. Middle: Wildcat Canyon (WC), second vs last year. Bottom: Vaughn Canyon (VC), first vs last year. Ctrl control, Tx treatment

Ordinations for all 500 bootstrap iterations were plotted and assessed for each site comparing treatment and control plots for the first and last year of observation, based on the assumption that the greatest difference would be found between the first and last year of observation. Representative ordinations are shown in Fig. 6; they were selected by summing the differences between the dissimilarity values in the ordination and the median dissimilarity values for all ordinations; the lowest summed difference was used as the representative ordination. At Barboot, there were shifts in composition at both treatment and control plots between 2015 and 2019 but with no clear directionality and overlap between groups. At Wildcat Canyon, ordinations were also created to compare the second and last year since two control plots were added in 2016. Plots with disparate single species present created outlier values that expanded ordination hulls, but both with and without these outliers there was no clear composition trend. Vaughn Canyon did not have enough plots to create ordination hulls; while some shifts in composition seem present, the lack of hulls makes determination difficult. Ordinations were also plotted comparing treatment and control plots for the first and last year of the study across all sites combined. In these ordinations, the diversity of the community at Barboot overwhelmed the other sites.

## Discussion

The frequency analysis showed a consistency in results within the bootstrapped iterations, but the species composition results were less straightforward. Perfectly similar plots (dissimilarity values of 0) occurred when both plots had no vegetation in the quadrats used for that bootstrap iteration. However, once sparse vegetation occurred, plot comparisons were often perfectly dissimilar (value of 1). This was due to the subset of quadrats in the bootstrap iteration for one plot capturing one individual plant while the subset for the other plot was empty or also captured one plant but of a different species. As the abundance of vegetation increased, the likelihood of empty subsets of quadrats for a plot decreased while the probability of shared species increased. Therefore, the changes in dissimilarity values may be due to differences in vegetation abundance rather than true differences in composition. The high occurrence of extreme dissimilarity values also limited the usefulness of the NMDS analysis since perfectly dissimilar values skewed the ordination, often excessively.

**Fig. 7:**
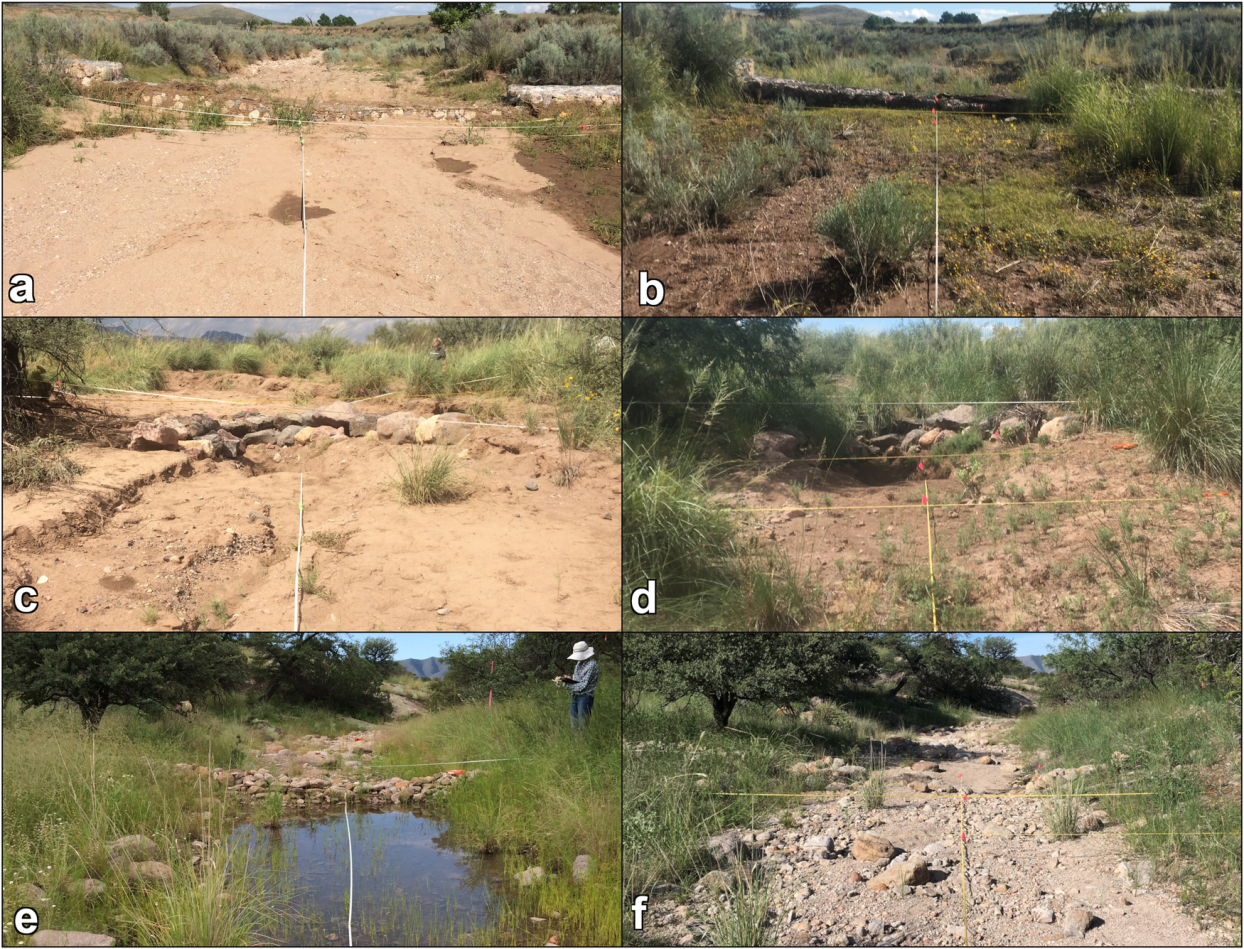
Repeat photographs of treatment plots from each site, the first and last year of the study. **a** Vaughn Canyon, 2015; looking downstream. **b** Vaughn Canyon, 2019; looking downstream. **c** Wildcat Canyon, 2015; looking upstream. **d** Wildcat Canyon, 2019; looking upstream. **e** Barboot, 2015; looking downstream. **f** Barboot, 2018; looking downstream. The RDS is full of sediment but still present. USGS photos by N. R. Wilson

At Barboot, frequency varied at plots over time as much as it did between treatment and control plots. In general, treatment plots had higher frequencies of vegetation, but since no before data were collected this could be an artifact of the restoration practitioners choosing RDS locations in areas where there was more vegetation present. More vegetation can lower risk of high flows that not only impact the RDS but also are a concern in the region. Two treatment plots, with very low frequencies of vegetation in 2015 were abandoned due to the scouring of the RDS during the 2016 monsoon. Alternatively, it may have been difficult to find non-vegetated, yet scour-protected locations for RDS at this site since it was the least degraded site. In species composition dissimilarity analysis, 62% of the 2015 dissimilarity values were extreme and 46% were extreme in 2018. This is a combination of the variability in vegetation abundance at the site as well as the higher species diversity. In both years there is substantial overlap in intergroup and intragroup dissimilarities. The ordination results show some variability between groups but it is not obviously due to treatment. Of the species found in quadrats most were shared between treatment and control plots. Only 4 were found exclusively at treatment plots while another 4 were found only at control plots. Additionally, in 2019 RDS from an undocumented preexisting restoration effort were found above the 2015 restoration project. Many of them had trapped sediment and well-established perennial vegetation growth within the rocks of the RDS indicating an extended presence on the landscape. Well-established restoration using RDS can support vegetation both upstream and downstream of the RDS locations (Wilson and Norman 2018). Given this, our project site at Barboot was likely already experiencing the effects of upstream RDS and was closer to its ecological potential than our other study sites. The fifth year of data collection was cancelled after discovering the upstream RDS might be affecting our study site and that preliminary analysis of the first 4 years of data showed no clear change in vegetation. However, the RDS may still meet the restoration goal of increasing resilience to catastrophic wildfire (Fig. 7).

At Wildcat Canyon, the frequency of perennial vegetation increased at treatment plots compared to control plots. The one treatment plot that did not have a clear increase was a RDS that was part of a constructed secondary channel to encourage braiding on the floodplain; the other treatment plots were in the constructed primary channel. The primary channel had clear evidence of regular flows (change in sediment size between years, debris lines, moist soil, etc.) during our observation period while the secondary channel did not. Without flows, RDS cannot slow flows, trap sediment, or provide other hydrological ecosystem services needed for the establishment and growth of vegetation. In the primary channel, the increased abundance could be due to *S. wrightii* being planted as plugs and larger relocations from local sources. While an effort was made to distinguish these plantings from natural recruitment during data collection, the efficacy of that was unclear and *S. wrightii* plants were not distinguished in analysis. However, plantings ceased before the 2016 field season. Any increase in frequency in 2017 and later would either be natural recruitment or the growth of successful plantings, to which the RDS may have contributed. In species composition analysis, 96% of the dissimilarities were extreme in 2015 and 79% of the values were still extreme in 2019. Vegetation abundance wasn’t high enough to make meaningfully intra- or inter- group comparisons quantitatively. This is evident in the NMDS ordination as treatment and control groups for each year have extreme outliers as well as overlaps with the other groups. The treatment plots did have 9 species not found at control plots, including 2 wetland species. One of those, *S. wrightii*, was planted at the sites during restoration. The other was *C. esculentus*, a weedy, facultative wetland perennial species that appeared at Wildcat Canyon and Vaughn Canyon treatment plots. At Wildcat Canyon, RDS did increase the abundance of perennial vegetation, if only by supporting plantings, but there was no clear shift in species composition (Fig. 7). At a site as degraded as Wildcat, it may take more time to see species composition change without intentional introduction via planting or seeding (Aavik and Helm 2018).

In the first year of the study at Vaughn Canyon, one treatment plot had much greater abundance of perennial vegetation than the other treatment plot or the control plots. This occurred after one monsoon season and primarily consisted of nonnative species. Though there was no quantitative data collected before RDS installation, anecdotal observation during the scoping period did not note that level of vegetation abundance in the channel. Also, the nonnative species that provide most of the vegetation frequency that first year were not observed at the project site at all prior to restoration. These nonnatives may have been introduced on the machinery used to install the RDS or may have colonized the location from nearby sources after the disturbance of the installation provided suitable habitat. At the other treatment plot, the first monsoon season damaged the RDS, undercutting the corners of the RDS and undermining its function. Repairs were made to the RDS during the second season of observation. Once the repairs were completed, perennial vegetation increased the following year. The RDS at Vaughn Canyon are large gabions which require heavy machinery to install and repair, a significant disturbance, which likely delayed the vegetation response at that plot. At Vaughn Canyon, control plots also had increases in perennial vegetation, particularly in the last two years of observation. Both control plots were downstream of other RDS and may have been experiencing downstream effects even in the short time period of 5 years. In species composition analysis, at Vaughn Canyon in 2015, 86% of the values were extreme. However, in 2019, only 6% of the values were extreme. Vegetation abundance had increased enough for meaningful species composition comparison between groups. Vaughn Canyon had only 4 plots, which made ordination inconclusive. However, the bootstrapped dissimilarity analysis shows that in the last year of the study, intergroup dissimilarity is higher than intragroup dissimilarity, indicating a difference in species composition between control and treatment plots at Vaughn Canyon. And in the quadrats, 14 species were only observed at treatment plots, 2 were nonnative perennial grasses and 3 were wetland species. The nonnatives included *S. halepense* and *C. dactylon*. For the wetland species, *C. esculentus* was present, along with an annual facultative wetland grass species, *Eriochloa acuminata* (tapertip cupgrass). *S. wrightii*, a facultative perennial bunchgrass, was also found in quadrats only at treatment plots, though it was one of the dominant species on the bank throughout the site. This indicates that the RDS created a habitat suitable for *S. wrightii* incursion into the stream channel. The restoration at Vaughn Canyon both increased vegetation abundance and shifted the species composition within the channel, though the shift included the introduction of nonnative as well as wetland species (Fig. 7).

Our results support our previous remote sensing research showing that RDS increases vegetation abundance (Norman et al. 2014; Wilson and Norman 2018). It extends those findings to show a trajectory of increased vegetation at RDS sites in degraded landscapes which doesn’t occur at intact sites. Largely, this increase is portrayed by increased abundance of existing vegetation, determined through our frequency and dissimilarity analyses. The increase in abundance was caused by the invasion of 2 nonnative species at one site (Vaughn Canyon), the introduction of 2 facultative wetland species at the same site, and the expansion of an existing wetland species at both degraded sites (Vaughn Canyon and Wildcat Canyon). At the least degraded site, no changes in abundance or composition were observed. Different types of RDS were used at each project site; however, due to the variability in baseline vegetation community and level of degradation, we draw no conclusions on the comparative efficacy of RDS types to increase vegetation abundance and diversity.

The invasion of nonnative species can be alarming, but the role of these species in restoration is not settled for restoration practitioners and stakeholders (D’Antonio and Meyerson 2002; Society for Ecological Restoration International Science & Policy Working Group 2004). Conventionally, the goal of a restoration project is to return the site to a prior state or to match a reference site, unaltered with nonnatives. However, for some practitioners, particularly when considering severely degraded sites, the goal is to return a site to a certain level of self-sustaining ecosystem function, typically defined as rehabilitation, or ecosystem restoration, instead of ecological restoration (Gann et al. 2019). As such, nativity of a species is less important than the functional profile of a species (Ewel and Putz 2004; Gornish et al. 2016). Therefore, if nonnative species are the primary components that meet the goal of a project (e.g., increased vegetation, stabilized soil, and/or reduced erosion), whether that is judged a success is based on of the values of the stakeholders.

Alternatively, wetland species are generally desired in riparian restoration and the increase in abundance anticipated, which occurred at both degraded sites. At the most intact, least degraded site, RDS had no clear effect because wetland species occurred at both treatment and control plots. In contrast, the facultative wetland bunchgrass, *S. wrightii*, drove the increase in vegetation abundance at the severely degraded Wildcat Canyon. At both Wildcat Canyon and Vaughn Canyon, *S. wrightii* occurred prior to restoration and at Wildcat Canyon it was planted as part of the restoration. However, it is likely that the success of those plantings at Wildcat Canyon was supported by the installation of RDS and at Vaughn Canyon RDS facilitated the movement of *S. wrightii* into the channel. Another wetland facultative species, the weedy perennial sedge *C. esculentus*, was found at all 3 sites, but it was found only at treatment plots at Vaughn Canyon. Vaughn Canyon also had another species, the wetland facultative annual grass *E. acuminata*, only at treatment plots. This greater increase in wetland vegetation species and the greater overall composition change at treatment plots at Vaughn Canyon might be because Vaughn Canyon is situated ~4 km upstream from the Babocomari Ciénega and 8 km north of the Canelo Hills Ciénega. Proximity to historic cienegas could indicate accessibility to groundwater and serve as nearby seed sources for these new species. Likewise, the increase in the abundance and receptivity of facultative wetland or facultative species at Wildcat Canyon could be affected by the extensive RDS installations that support the health of Ciénega San Bernardino, 3 km downstream (Norman et al. 2014). Our less responsive Barboot site is located 11 km upstream from the Leslie Canyon Ciénega and therefore, likely less connected. Minckley et al. (2013) documented that flow of subsurface water around historic ciénegas is distributed laterally and longitudinally, allowing ciénegas to extend 100 s of meters and suggest threats associated with groundwater overdrafts that disconnect surface waters with the water table and root zone cause ciénega decline. In research done at the Vaughn Canyon site, Norman et al. (2019) documented increasing lateral flows associated with overbank flooding of subsurface flow around RDS. Research done at the San Bernardino ciénega depicted increased vegetation at areas up to 1 km upstream and 5 km downstream of RDS (Wilson and Norman 2018). The proximity of intact ciénegas and other riparian habitats may increase the efficacy of RDS to restore historic ciénegas and may, with more time, create new wetland-like environments in dryland streams that are seasonally or permanently saturated with water (Norman et al. 2022b).

### Challenges

Natural and human disturbances in the systems created challenges for assessing the effectiveness of the treatments. For example, two project sites had flows that damaged RDS and required repair. We also had data from two project sites that we could not use due to private landowner actions. One site was unusable because the landowner continued restoration efforts on study plots over the 5 years of observation and the second site was unusable because the landowner shifted focus to another section of land, after we had collected baseline data. On a broader scale, the spatially heterogenous summer monsoon rains of the Madrean Archipelago can impact the efficacy of restoration research. RDS are built to affect stream flow, if there is no flow during a period of time, the effects of RDS cannot be analyzed. On the other hand, several years of higher-than-average rains can allow early successional wetland species to appear in areas where they won’t persist during normal years. Thankfully, our research was not affected by either of these extremes. Additionally, project planning constraints can limit the study design by providing inadequate time to identify suitable reference or control locations and preventing data collection prior to the implementation of restoration. Another challenge is balancing the time needed to collect sufficiently detailed data against the time needed to collect a suitable sample size, further complicated by the need for repeated sampling for additional time steps.

A challenge unique to the assessment of watershed restoration projects which most conventional restoration projects avoid is the possibility of differing vegetation response across a complex topography. Anecdotal evidence from mature restoration sites as well as ecohydrological concepts supported by our research indicate vegetation response would vary across topographic positions, including within the channel, along the bank, and on the floodplain, necessitating development of a hybrid approach. Here we presented the results of the short-term (0–5 years), in-channel study. We have also collected preliminary data from the floodplain and banks adjacent to the channel which could allow analysis of long-term, decadal changes.

### Lessons Learned

We have many lessons learned from this multi-year project to share and employ in future research. One of the easiest changes would be to standardize and monument the extent of the zones with permanent rebar markers to allow for more quantitative analyses and better document the changes through time and space. Ideally, baseline data would be collected before RDS were installed would allow for a full Before-After-Control-Impact-Paired-Series (BACIPS) design (Underwood 1994; Thiault et al. 2017). In our previous research on large RDS (gabions), we found that, over a period of decades, the entire length of restored channel and beyond is affected (Norman et al. 2014; Wilson and Norman 2018). Therefore, an improvement to our current design would be to better locate controls outside of the restored channel, such as with a paired watershed analysis design which matches approximate drainage area, restored length, channel morphology, vegetation community, etc. (Petrakis et al. 2021). Additionally, optimal data collection would occur during each growing season (Underwood 1994). For our study area, that means including the small winter/spring growing season in addition to the monsoonal summer growing season which was the focus of our data collection. This investigation into the spring growing season could be completed with remote sensing. A large component of both growing seasons are annual plants, which were highly generalized in this study. A greater investigation into annual plant species composition and abundance could help us understand the successional trajectory site and combined with habitat provisioning analysis how a site is used by wildlife. RDS are known for capturing larger or heavier sediment in surface-runoff. There were some noticeable changes in sediment size between years, particularly at Wildcat Canyon, which could be due to either upstream sediment dropout or small tributary source watershed landscape contributing to the mixture. The addition of soil particle size analysis would better define the state of the RDS and response in the watershed. This, combined with carbon flux analysis of both soils and vegetation are our next steps locally. Finally, related to hydroclimatic systems, the integration of soil moisture, temperature, and precipitation measurements, in the field or via remote sensing, would situate the findings of this research in a larger climatic context.

## Conclusion

Repeat field surveys at sites where rock detention structures (RDS) were installed provide new insight into the effects RDS have on vegetation. Our results document that RDS can (i) increase vegetation abundance at degraded sites, (ii) introduce nonnatives at sites, and (iii) may provide the ecohydrologic conditions for new facultative wetland species to appear and existing wetland species to expand. We find that sites in close proximity to historic or active ciénegas are more responsive to restoration treatments, which suggests improved hydrological connectivity. Our least degraded, closest to baseline site saw little change from the installation of RDS. Our most degraded site, Wildcat Canyon, did respond to RDS, indicating that it was not past an ecological threshold though that response was less robust than that at Vaughn Canyon. Vaughn Canyon was less degraded than Wildcat Canyon and responded strongly to RDS. Our findings agree with the growing literature on the application of ecological threshold theory to restoration; a landscape that is degraded to some degree, but not past an ecological threshold, is likely to benefit most from restoration and revegetation efforts (Buisson et al. 2019; Lindenmayer 2020).

## Supplementary Information

Supplementary Materials

## Data Availability

All data collected as part of this study are publicly available on USGS’s ScienceBase, 10.5066/P9ED4O3K.
