# Supplementary material for: Five Year Analyses of Vegetation Response to Restoration using Rock Detention Structures in Southeastern Arizona, United States
Source: Environ Manage. 2022 Dec 19;71(5):921–39. doi: 10.1007/s00267-022-01762-0 (PMC10083153; doi:10.1007/s00267-022-01762-0)
Supplement: Supplementary file 1 — Supplementary Materials [file 267_2022_1762_MOESM1_ESM.docx]

# Five Year Analyses of Vegetation Response to Restoration using Rock Detention Structures in Southeastern Arizona, United States

# Supplementary Materials

**Journal**: Environmental Management

**Authors**: Natalie R. Wilson^1^ (ORCID: 0000-0001-5145-1221) and Laura M. Norman^1^ (ORCID: 0000-0002-3696-8406)

**Author affiliations**: ^1^U.S. Geological Survey, Western Geographic Science Center, 520 N. Park Ave, Tucson, Arizona 85719

**Corresponding author**: Natalie R. Wilson, [nrwilson@usgs.gov](mailto:nrwilson@usgs.gov)

# Figures


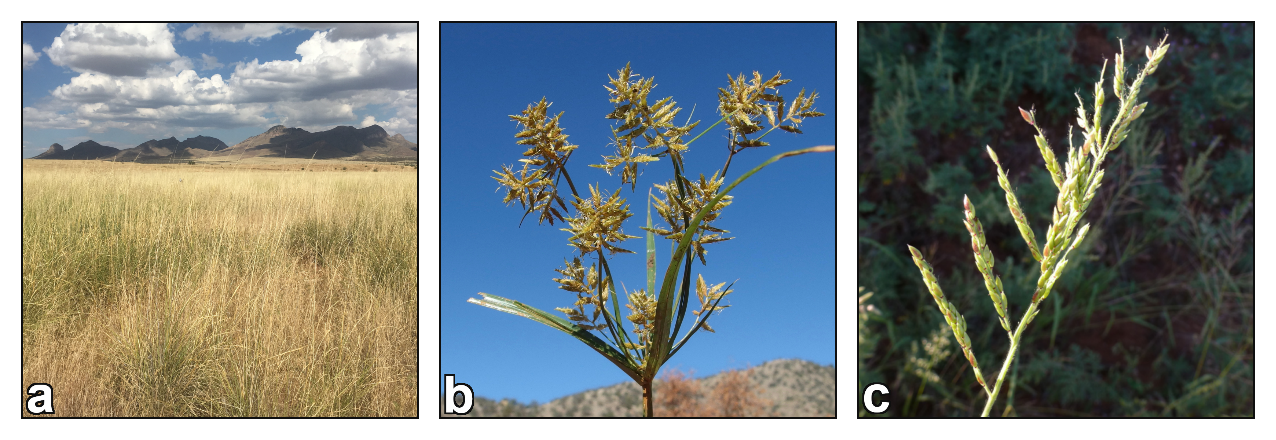


**Fig S1** Wetland plant species found at our study sites. a) Facultative species *Sporobolus wrightii*, giant sacaton. Photo credit: Natalie R. Wilson. b) Facultative wetland species *Cyperus esculentus*, yellow nutsedge. Photo credit: Sue Carnahan. c) Facultative wetland species *Eriochloa acuminata*, tapertip cupgrass. Photo credit: Max Licher. Photos b and c retrieved from SEINet, <https://swbiodiversity.org/seinet>, accessed 6/25/22

# Tables

Table S1. Species observed in quadrats. Separated into Control (Ctrl), Treatment (Tx), and entire site (All).

| Family | Species | Barboot | | | Wildcat Canyon | | | Vaughn Canyon | | |
| --- | --- | --- | --- | --- | --- | --- | --- | --- | --- | --- |
|  |  | Ctrl | Tx | All | Ctrl | Tx | All | Ctrl | Tx | All |
| Apiaceae | *Eryngium heterophyllum†* |  | X | X |  |  |  |  |  |  |
| Aristolochiaceae | *Aristolochia watsonii* |  |  |  |  | X | X |  |  |  |
| Asteraceae | *Ambrosia confertiflora* |  |  |  | X | X | X |  | X | X |
|  | *Ambrosia monogyra* |  |  |  | X |  | X |  |  |  |
|  | *Artemisia ludoviciana* | X | X | X |  |  |  |  |  |  |
|  | *Erigeron divergens* | X | X | X |  |  |  |  |  |  |
|  | *Ericameria nauseosa* |  |  |  |  |  |  | X | X | X |
|  | *Flourensia cernua* |  |  |  |  | X | X |  |  |  |
|  | *Machaeranthera pinnatifida* |  |  |  |  | X | X |  | X | X |
|  | *Pseudognaphalium* spp | X |  | X |  |  |  |  |  |  |
| Commelinaceae | *Commelina dianthifolia* |  | X | X |  |  |  |  |  |  |
| Cyperaceae | *Cyperus esculentus†* | X | X | X |  | X | X |  | X | X |
|  | *Cyperus pallidicolor/hermaphroditus* complex | X |  | X |  |  |  |  | X | X |
|  | *Cyperus sphaerolepis* |  | X | X |  |  |  |  |  |  |
|  | *Perennial sedge* sp |  |  |  | X |  | X |  |  |  |
| Euphorbiaceae | *Acalypha phleoides* |  |  |  |  |  |  |  | X | X |
| Fabaceae | *Calliandra humilis* |  | X | X |  |  |  |  |  |  |
|  | *Galactia wrightii* |  | X | X |  |  |  |  |  |  |
|  | *Macroptilium gibbosifolium* |  | X | X |  |  |  |  |  |  |
|  | *Prosopis velutina* |  |  |  | X | X | X |  |  |  |
|  | *Rhynchosia senna* |  | X | X |  |  |  |  |  |  |
|  | *Vachellia constricta* |  |  |  |  | X | X |  |  |  |
| Liliaceae | *Nolina microcarpa* |  | X | X |  |  |  |  |  |  |
| Malvaceae | *Sphaeralcea angustifolia* |  |  |  |  |  |  |  | X | X |
| Nyctaginaceae | *Boerhavia coccinea* |  |  |  |  | X | X |  | X | X |
|  | *Mirabilis longiflora* | X |  | X |  |  |  |  |  |  |
| Onagraceae | *Epilobium canum* |  | X | X |  |  |  |  |  |  |
|  | *Oenothera* spp |  | X | X |  |  |  |  |  |  |
| Papaveraceae | *Argemone pleiacantha* |  |  |  |  |  |  | X | X | X |
| Poaceae | *Aristida ternipes* | X | X | X |  | X | X | X | X | X |
|  | *Bothriochloa barbinodis* | X | X | X |  |  |  |  |  |  |
|  | *Bouteloua curtipendula* | X | X | X |  |  |  | X | X | X |
|  | *Bouteloua hirsuta* |  | X | X |  |  |  |  |  |  |
|  | *Bouteloua radicosa* | X | X | X |  |  |  |  |  |  |
|  | *Cynodon dactylon** |  |  |  |  |  |  |  | X | X |
|  | *Digitaria californica* |  |  |  |  | X | X |  |  |  |
|  | *Eragrostis intermedia* | X | X | X |  |  |  |  |  |  |
|  | *Eragrostis lehmanniana* | X | X | X |  |  |  | X | X | X |
|  | *Leptochloa dubia* | X | X | X |  |  |  |  | X | X |
|  | *Lycurus phleoides* | X |  | X |  |  |  |  |  |  |
|  | *Lycurus setosus* | X | X | X |  |  |  |  |  |  |
|  | *Muhlenbergia emersleyi* | X | X | X |  |  |  |  |  |  |
|  | *Muhlenbergia palmeri* | X |  | X |  |  |  |  |  |  |
|  | *Muhlenbergia rigens* | X | X | X |  |  |  |  | X | X |
|  | *Panicum bulbosum* |  | X | X |  |  |  |  |  |  |
|  | *Panicum obtusum* |  |  |  |  |  |  |  | X | X |
|  | *Schizachyrium cirratum/sanguineum* complex | X | X | X |  |  |  |  |  |  |
|  | *Setaria leucopila/macrostachys* complex |  |  |  |  | X | X |  |  |  |
|  | *Sorghum halepense** |  |  |  | X |  | X |  | X | X |
|  | *Sporobolus contractus* |  |  |  |  |  |  |  | X | X |
|  | *Sporobolus cryptandrus* |  |  |  |  |  |  | X | X | X |
|  | *Sporobolus wrightii†* |  |  |  |  | X | X |  | X | X |
| Polemoniaceae | *Ipomopsis macombii* |  | X | X |  |  |  |  | X | X |
| Solanaceae | *Solanum elaeagnifolium* |  |  |  |  | X | X |  |  |  |
| ** indicates nonnative species*  *† indicates wetland species* | | | | | | | | | | |

Table S2. Species observed in zones. Separated into Control (Ctrl), Treatment (Tx), and entire site (All).

| Family | *Species* | Barboot | | | Wildcat | | | Vaughn Canyon | | |  |
| --- | --- | --- | --- | --- | --- | --- | --- | --- | --- | --- | --- |
|  |  | Ctrl | Tx | All | Ctrl | Tx | All | Ctrl | Tx | All |  |
| Acanthaceae | *Carlowrightia arizonica* |  |  |  |  | X | X |  |  |  |  |
|  | *Dyschoriste schiedeana* |  | X | X |  |  |  |  |  |  |  |
|  | *Ruellia nudiflora* |  |  |  | X | X | X |  |  |  |  |
| Agavaceae | *Agave palmeri* | X | X | X |  |  |  |  |  |  |  |
|  | *Yucca baccata* |  | X | X |  |  |  |  |  |  |  |
|  | *Yucca elata* |  |  |  |  | X | X | X |  | X |  |
|  | *Yucca madrensis* |  | X | X |  |  |  |  |  |  |  |
| Amaranthaceae | *Froelichia arizonica* |  |  |  |  |  |  | X |  | X |  |
|  | *Guilleminea densa* | X | X | X |  |  |  | X | X | X |  |
| Anacardiaceae | *Rhus microphylla* |  |  |  | X | X | X | X |  | X |  |
|  | *Rhus trilobata* |  |  |  | X |  | X |  |  |  |  |
| Apiaceae | *Eryngium heterophyllum†* | X | X | X |  |  |  |  |  |  |  |
| Aristolochiaceae | *Aristolochia watsonii* | X |  | X | X | X | X |  |  |  |  |
| Asclepiadaceae | *Asclepias glaucescens* |  | X | X |  |  |  |  |  |  |  |
|  | *Asclepias nyctaginifolia* | X |  | X |  |  |  |  |  |  |  |
|  | *Funastrum cynanchoides* |  |  |  | X |  | X |  |  |  |  |
|  | *Matelea producta* |  |  |  | X |  | X |  |  |  |  |
| Asteraceae | *Ambrosia confertiflora* | X |  | X | X | X | X | X | X | X |  |
|  | *Ambrosia monogyra* |  |  |  | X | X | X |  |  |  |  |
|  | *Artemisia ludoviciana* | X | X | X |  |  |  | X |  | X |  |
|  | *Baccharis emoryi†* |  |  |  | X |  | X |  |  |  |  |
|  | *Baccharis pteronioides* |  |  |  |  |  |  | X | X | X |  |
|  | *Baccharis sarothroides* |  |  |  | X | X | X |  |  |  |  |
|  | *Baccharis thesioides* | X |  | X |  |  |  |  |  |  |  |
|  | *Berlandiera lyrata* |  |  |  |  |  |  | X | X | X |  |
|  | *Brickellia californica* | X | X | X |  |  |  |  |  |  |  |
|  | *Brickellia eupatorioides* |  | X | X |  |  |  |  | X | X |  |
|  | *Brickellia floribunda†* |  |  |  | X |  | X | X | X | X |  |
|  | *Brickellia venosa* | X | X | X |  |  |  |  |  |  |  |
|  | *Carphochaete bigelovii* |  | X | X |  |  |  |  |  |  |  |
|  | *Cirsium ochrocentrum* |  |  |  |  |  |  |  | X | X |  |
|  | *Cirsium undulatum* |  |  |  |  |  |  |  | X | X |  |
|  | *Ericameria nauseosa* |  |  |  |  |  |  | X | X | X |  |
|  | *Erigeron divergens* | X | X | X |  |  |  |  |  |  |  |
|  | *Erigeron flagellaris* |  |  |  |  |  |  | X | X | X |  |
|  | *Erigeron neomexicanus* | X | X | X |  |  |  |  |  |  |  |
|  | *Flourensia cernua* |  |  |  |  | X | X |  |  |  |  |
|  | *Gaillardia pinnatifida* | X |  | X |  |  |  |  |  |  |  |
|  | *Gutierrezia microcephala* |  |  |  | X | X | X |  |  |  |  |
|  | *Heliomeris multiflora* | X |  | X |  |  |  |  |  |  |  |
|  | *Isocoma tenuisecta* |  |  |  | X | X | X | X |  | X |  |
|  | *Lasianthaea podocephala* | X |  | X |  |  |  |  |  |  |  |
|  | *Machaeranthera pinnatifida* |  |  |  |  | X | X | X | X | X |  |
|  | *Pseudognaphalium* spp | X |  | X |  |  |  |  |  |  |  |
|  | *Thelesperma megapotamicum* | X |  | X |  |  |  | X | X | X |  |
|  | *Viguiera cordifolia* | X | X | X |  |  |  |  |  |  |  |
|  | *Viguiera dentata* | X | X | X | X |  | X | X | X | X |  |
|  | *Viguiera parishii* |  |  |  |  |  |  | X |  | X |  |
|  | *Zinnia grandiflora* |  | X | X |  |  |  |  |  |  |  |
| Boraginaceae | *Lithospermum* spp |  |  |  |  |  |  | X |  | X |  |
| Brassicaceae | *Pennellia micrantha* |  | X | X |  |  |  |  |  |  |  |
|  | *Schoenocrambe linearifolia* | X | X | X |  |  |  | X | X | X |  |
| Cactaceae | *Cylindropuntia spinosior* | X | X | X |  |  |  |  |  |  |  |
| Capparaceae | *Cleome* spp |  |  |  |  |  |  | X | X | X |  |
| Chenopodiaceae | *Atriplex canescens* |  |  |  | X | X | X |  |  |  |  |
| Commelinaceae | *Commelina dianthifolia* | X | X | X |  |  |  |  |  |  |  |
|  | *Commelina erecta* |  |  |  | X |  | X |  |  |  |  |
|  | *Commelina* spp |  |  |  |  |  |  |  | X | X |  |
|  | *Tradescantia pinetorum* | X |  | X |  |  |  |  |  |  |  |
| Convolvulaceae | *Evolvulus arizonicus* | X |  | X |  |  |  |  |  |  |  |
|  | *Evolvulus sericeus* | X |  | X |  |  |  |  |  |  |  |
| Cucurbitaceae | *Apodanthera undulata* |  |  |  |  | X | X |  |  |  |  |
|  | *Cucurbita digitata* |  |  |  | X |  | X | X | X | X |  |
| Cupressaceae | *Juniperus deppeana* | X |  | X |  |  |  |  |  |  |  |
|  | *Juniperus monosperma* | X | X | X |  |  |  |  |  |  |  |
| Cyperaceae | *Cyperus dipsaceus* | X | X | X |  |  |  |  |  |  |  |
|  | *Cyperus esculentus†* | X | X | X | X | X | X | X | X | X |  |
|  | *Cyperus pallidicolor/hermaphroditus* complex | X | X | X |  |  |  |  | X | X |  |
|  | *Cyperus sphaerolepis* | X | X | X |  |  |  |  |  |  |  |
| Ephedraceae | *Ephedra trifurca* |  |  |  | X |  | X |  |  |  |  |
| Ericaceae | *Arctostaphylos pungens* | X | X | X |  |  |  |  |  |  |  |
| Euphorbiaceae | *Acalypha phleoides* | X |  | X |  |  |  |  | X | X |  |
|  | *Chamaesyce albomarginata* |  |  |  |  | X | X |  |  |  |  |
|  | *Croton pottsii* |  |  |  |  |  |  | X |  | X |  |
|  | *Jatropha macrorhiza* | X | X | X |  |  |  | X | X | X |  |
|  | *Tragia nepetifolia* |  | X | X |  |  |  |  |  |  |  |
| Fabaceae | *Acaciella angustissima* |  | X | X |  |  |  |  |  |  |  |
|  | *Astragalus* spp | X |  | X |  |  |  |  |  |  |  |
|  | *Calliandra humilis* | X | X | X |  |  |  |  |  |  |  |
|  | *Cologania angustifolia* | X | X | X |  |  |  |  |  |  |  |
|  | *Dalea albiflora* | X | X | X |  |  |  |  |  |  |  |
|  | *Dalea grayi* | X | X | X |  |  |  |  |  |  |  |
|  | *Desmodium batocaulon* | X |  | X |  |  |  |  |  |  |  |
|  | *Galactia wrightii* | X | X | X |  |  |  |  |  |  |  |
|  | *Hoffmannseggia drepanocarpa* |  |  |  |  | X | X |  |  |  |  |
|  | *Hoffmannseggia glauca* |  |  |  |  | X | X |  |  |  |  |
|  | *Macroptilium gibbosifolium* | X | X | X |  |  |  |  |  |  |  |
|  | *Mimosa aculeaticarpa* | X | X | X |  |  |  |  |  |  |  |
|  | *Mimosa dysocarpa* | X |  | X |  |  |  |  |  |  |  |
|  | *Phaseolus ritensis* |  | X | X |  |  |  |  |  |  |  |
|  | *Prosopis velutina* | X | X | X | X | X | X | X | X | X |  |
|  | *Psoralidium tenuiflorum* | X |  | X |  |  |  |  | X | X |  |
|  | *Rhynchosia senna* | X | X | X | X |  | X |  | X | X |  |
|  | *Vachellia constricta* |  |  |  | X | X | X |  |  |  |  |
| Fagaceae | *Quercus emoryi* | X | X | X |  |  |  |  |  |  |  |
|  | *Quercus grisea* | X | X | X |  |  |  |  |  |  |  |
|  | *Quercus toumeyi* | X | X | X |  |  |  |  |  |  |  |
|  | *Quercus turbinella* |  | X | X |  |  |  |  |  |  |  |
| Garryaceae | *Garrya wrightii* |  | X | X |  |  |  |  |  |  |  |
| Lamiaceae | *Trichostema arizonicum* | X | X | X |  |  |  |  |  |  |  |
| Liliaceae | *Milla biflora* | X | X | X |  |  |  |  |  |  |  |
|  | *Nolina microcarpa* | X | X | X |  |  |  |  |  |  |  |
| Loasaceae | *Mentzelia multiflora* |  |  |  |  |  |  |  | X | X |  |
| Malpighiaceae | *Aspicarpa hirtella* | X | X | X |  |  |  |  |  |  |  |
|  | *Janusia gracilis* |  |  |  |  | X | X |  |  |  |  |
| Malvaceae | *Abutilon parvulum* |  |  |  | X | X | X |  |  |  |  |
|  | *Rhynchosida physocalyx* |  |  |  | X | X | X | X | X | X |  |
|  | *Sphaeralcea* spp | X | X | X |  |  |  |  |  |  |  |
|  | *Sphaeralcea angustifolia* |  |  |  | X | X | X | X | X | X |  |
| Nyctaginaceae | *Boerhavia coccinea* | X | X | X | X | X | X | X | X | X |  |
|  | *Mirabilis coccinea* | X |  | X |  |  |  |  |  |  |  |
|  | *Mirabilis linearis* | X | X | X |  |  |  |  |  |  |  |
|  | *Mirabilis longiflora* | X | X | X |  |  |  |  |  |  |  |
| Onagraceae | *Epilobium canum* | X | X | X |  |  |  |  |  |  |  |
|  | *Oenothera flava* |  |  |  |  |  |  |  | X | X |  |
|  | *Oenothera suffrutescens* | X | X | X |  |  |  | X | X | X |  |
| Papaveraceae | *Argemone pleiacantha* |  |  |  |  |  |  | X | X | X |  |
| Pedaliaceae | *Proboscidea althaeifolia* |  |  |  |  | X | X |  |  |  |  |
|  | *Proboscidea* spp |  | X | X |  |  |  |  |  |  |  |
| Poaceae | *Aristida schiedeana* | X | X | X |  |  |  |  |  |  |  |
|  | *Aristida ternipes* | X | X | X |  | X | X | X | X | X |  |
|  | *Bothriochloa barbinodis* | X | X | X | X | X | X | X | X | X |  |
|  | *Bothriochloa ischaemum* |  |  |  |  |  |  | X |  | X |  |
|  | *Bothriochloa laguroides* |  |  |  |  |  |  |  | X | X |  |
|  | *Bouteloua curtipendula* | X | X | X | X |  | X | X | X | X |  |
|  | *Bouteloua eriopoda* | X |  | X |  |  |  | X |  | X |  |
|  | *Bouteloua gracilis* | X | X | X |  |  |  | X | X | X |  |
|  | *Bouteloua hirsuta* | X | X | X |  |  |  | X | X | X |  |
|  | *Bouteloua radicosa* | X | X | X |  |  |  |  |  |  |  |
|  | *Bouteloua rothrockii* |  |  |  |  | X | X |  |  |  |  |
|  | *Cynodon dactylon** |  |  |  |  | X | X | X | X | X |  |
|  | *Dasyochloa pulchella* |  |  |  |  | X | X |  |  |  |  |
|  | *Digitaria californica* |  |  |  | X | X | X |  |  |  |  |
|  | *Elymus elymoides* | X | X | X |  |  |  | X | X | X |  |
|  | *Enneapogon desvauxii* |  |  |  |  |  |  |  | X | X |  |
|  | *Eragrostis curvula* | X | X | X |  |  |  | X |  | X |  |
|  | *Eragrostis intermedia* | X | X | X |  | X | X |  |  |  |  |
|  | *Eragrostis lehmanniana* | X | X | X | X |  | X | X | X | X |  |
|  | *Heteropogon contortus* | X | X | X |  | X | X |  |  |  |  |
|  | *Hilaria belangeri* |  |  |  |  |  |  | X |  | X |  |
|  | *Koeleria macrantha* | X |  | X |  |  |  |  |  |  |  |
|  | *Leptochloa dubia* | X | X | X | X |  | X | X | X | X |  |
|  | *Lycurus phleoides* | X | X | X |  |  |  |  |  |  |  |
|  | *Lycurus setosus* | X | X | X |  |  |  |  |  |  |  |
|  | *Muhlenbergia dubia* | X |  | X |  |  |  |  |  |  |  |
|  | *Muhlenbergia emersleyi* | X | X | X |  |  |  |  |  |  |  |
|  | *Muhlenbergia longiligula* |  | X | X |  |  |  |  |  |  |  |
|  | *Muhlenbergia palmeri* | X |  | X |  |  |  |  |  |  |  |
|  | *Muhlenbergia pauciflora* | X |  | X |  |  |  |  |  |  |  |
|  | *Muhlenbergia porteri* |  |  |  | X | X | X |  |  |  |  |
|  | *Muhlenbergia repens* |  |  |  |  |  |  |  | X | X |  |
|  | *Muhlenbergia rigens* | X | X | X |  |  |  | X |  | X |  |
|  | *Panicum antidotale* | X | X | X |  |  |  |  |  |  |  |
|  | *Panicum bulbosum* | X | X | X |  |  |  |  |  |  |  |
|  | *Panicum obtusum* | X |  | X | X |  | X |  | X | X |  |
|  | *Panicum virgatum†* | X |  | X |  |  |  |  |  |  |  |
|  | *Pennisetum ciliare** |  |  |  | X |  | X |  |  |  |  |
|  | *Piptochaetium fimbriatum* | X |  | X |  |  |  |  |  |  |  |
|  | *Schizachyrium cirratum/sanguineum* complex | X | X | X |  |  |  |  |  |  |  |
|  | *Setaria leucopila/macrostachya* complex | X |  | X | X | X | X |  |  |  |  |
|  | *Sorghum halepense** | X | X | X | X | X | X | X | X | X |  |
|  | *Sporobolus contractus* |  |  |  |  |  |  | X | X | X |  |
|  | *Sporobolus cryptandrus* | X |  | X |  |  |  | X | X | X |  |
|  | *Sporobolus wrightii†* |  |  |  | X | X | X | X | X | X |  |
|  | *Trachypogon spicatus* | X | X | X |  |  |  |  |  |  |  |
| Polemoniaceae | *Ipomopsis macombii* | X | X | X |  |  |  | X | X | X |  |
| Polygalaceae | *Polygala alba* |  |  |  |  |  |  | X |  | X |  |
|  | *Polygala obscura* | X | X | X |  |  |  |  |  |  |  |
| Polygonaceae | *Eriogonum wrightii* | X | X | X |  |  |  |  |  |  |  |
|  | *Rumex hymenosepalus* |  |  |  | X | X | X |  |  |  |  |
| Portulacaceae | *Phemeranthus aurantiacus* | X | X | X |  |  |  | X |  | X |  |
|  | *Phemeranthus parvulus* | X |  | X |  |  |  |  |  |  |  |
|  | *Portulaca suffrutescens* |  |  |  |  |  |  |  | X | X |  |
|  | *Talinum paniculatum* | X | X | X |  |  |  |  |  |  |  |
| Pteridaceae | *Bommeria hispida* |  | X | X |  |  |  |  |  |  |  |
|  | *Cheilanthes eatonii* | X |  | X |  |  |  |  |  |  |  |
|  | *Cheilanthes fendleri* | X | X | X |  |  |  |  |  |  |  |
|  | *Cheilanthes lindheimeri* | X | X | X |  |  |  |  |  |  |  |
|  | *Cheilanthes wootonii* | X |  | X |  |  |  |  |  |  |  |
|  | *Pellaea truncata/wrightiana* complex | X | X | X |  |  |  |  |  |  |  |
|  | *Pellaea ternifolia* |  | X | X |  |  |  |  |  |  |  |
| Rubiaceae | *Galium microphyllum* | X |  | X |  |  |  |  |  |  |  |
|  | *Houstonia wrightii* | X |  | X |  |  |  |  |  |  |  |
| Salicaceae | *Populus fremontii†* |  |  |  |  |  |  | X |  | X |  |
|  | *Salix gooddingii†* |  |  |  |  |  |  | X |  | X |  |
| Scrophulariaceae | *Castilleja* spp | X |  | X |  |  |  |  |  |  |  |
|  | *Penstemon parryi* |  |  |  |  |  |  |  | X | X |  |
| Solanaceae | *Solanum elaeagnifolium* | X |  | X | X | X | X |  | X | X |  |
|  | *Solanum jamesii* |  |  |  |  |  |  |  | X | X |  |
| Ulmaceae | *Celtis laevigata var. reticulata* |  |  |  | X |  | X |  |  |  |  |
| Verbenaceae | *Aloysia wrightii* |  | X | X | X |  | X |  |  |  |  |
|  | *Glandularia bipinnatifida* | X |  | X |  |  |  | X | X | X |  |
|  | *Glandularia gooddingii* | X | X | X | X | X | X |  |  |  |  |
|  | *Tetraclea coulteri* |  |  |  |  |  |  |  | X | X |  |
|  | *Verbena neomexicana* | X |  | X |  |  |  |  |  |  |  |
| Violaceae | *Hybanthus verticillatus* |  |  |  |  | X | X |  | X | X |  |
| Vitaceae | *Vitis arizonica* | X | X | X |  |  |  |  |  |  |  |
| ** indicates nonnative species*  *† indicates wetland species* | | | | | | | | | | | |
